# Iron Regulation: Macrophages in Control

**DOI:** 10.3390/ph11040137

**Published:** 2018-12-14

**Authors:** Nyamdelger Sukhbaatar, Thomas Weichhart

**Affiliations:** Medical University of Vienna, Center for Pathobiochemistry and Genetics, Vienna 1090, Austria; nyamdelger.sukhbaatar@meduniwien.ac.at

**Keywords:** macrophage, central nurse macrophage, red pulp macrophage, Kupffer cell, iron metabolism, erythropoiesis, erythroblastic islands, erythrophagocytosis, inflammation

## Abstract

Macrophages are sentinel cells of the innate immune system and have important functions in development, tissue homeostasis, and immunity. These phylogenetically ancient cells also developed a variety of mechanisms to control erythropoiesis and the handling of iron. Red pulp macrophages in the spleen, Kupffer cells in the liver, and central nurse macrophages in the bone marrow ensure a coordinated metabolism of iron to support erythropoiesis. Phagocytosis of senescent red blood cells by macrophages in the spleen and the liver provide a continuous delivery of recycled iron under steady-state conditions and during anemic stress. Central nurse macrophages in the bone marrow utilize this iron and provide a cellular scaffold and niche to promote differentiation of erythroblasts. This review focuses on the role of the distinct macrophage populations that contribute to efficient iron metabolism and highlight important cellular and systemic mechanisms involved in iron-regulating processes.

## 1. Introduction

Iron is a multifaceted metal that plays a versatile role in many basic cellular processes in the body, including DNA synthesis, cellular metabolism and respiration, as well as cell growth and death mechanisms through iron-containing and iron-sequestering proteins and enzymes [1,2]. Moreover, iron is the main component of hemoglobin (Hgb) and is thus essential for oxygen transport within erythrocytes. As a redox-active metal, it can be found in two main oxidation states in the body that determine its features, functional roles, and toxicity. Whereas divalent ferrous iron (Fe^2+^) is the active compound in Hgb and in many enzymes, it can cause cellular toxicity by means of intracellular free radical production by Fenton reactions [3,4]. Therefore, iron is usually intracellularly stored or systemically transported as oxidized trivalent ferric iron (Fe^3+^). Iron metabolism is tightly regulated by a variety of redundant mechanisms that are often conserved throughout the mammalian kingdom to adjust iron concentrations on systemic and cellular levels. In response to iron and red blood cell (RBC) demand, an orchestrated interplay between iron-processing cells, including tissue macrophages, hepatocytes, erythrocytes, and duodenal epithelial cells controls and maintains iron homeostasis. In particular, macrophage populations play a key role as cellular components of iron metabolism to maintain the balance between the availability of sufficient iron levels and the prevention of toxic amounts of iron in the body. Tissue macrophages specialized for iron recycling include red pulp macrophages (RPMs) in the spleen, central nurse macrophages in bone marrow (BM), and Kupffer cells (KCs) in the liver. These macrophage populations have a tremendous ability to recycle between 90–95% of bodily iron and to maintain efficient erythropoiesis. Distortions in the equilibrium of iron demand and supply are sensed and corrected by a network of macrophages in their niches. 

Erythrophagocytic macrophages that are in direct contact with labile heme and iron developed protective mechanisms to cope with their toxicity. On the contrary, erythropoiesis requires a continuous delivery of iron, supplied presumably through BM macrophages. In cases of blood loss, menstrual bleeding, or pregnancy, a higher iron requirement is adjusted by increasing the intestinal absorption, the only iron-regulatory process in which a role for macrophages is currently unknown. Recent observations, however, suggested a potential role of intestinal macrophages to transiently store iron [5]. Vertebrates do not possess active iron secretory mechanisms, but mainly lose iron due to shedding and renewing of the intestinal cell layer. Distinct types of macrophages control iron-processing mechanisms in interplay with other cellular components and environmental cues. In fact, in adipose tissues a macrophage population with a high iron recycling gene expression profile was identified [6]. In obesity, this macrophage population shows an impaired ability to handle iron and adipogenesis is linked to tissue iron overload [6,7,8]. These findings suggest the possible general importance of macrophages in tissue iron metabolism and the role of iron as a link to metabolic disease. 

Here, we review the current knowledge of the cellular processes involved in iron metabolism and the regulatory mechanisms in iron-processing macrophages.

## 2. Cellular Uptake and Metabolism of Iron in Macrophages

**Figure 1:**
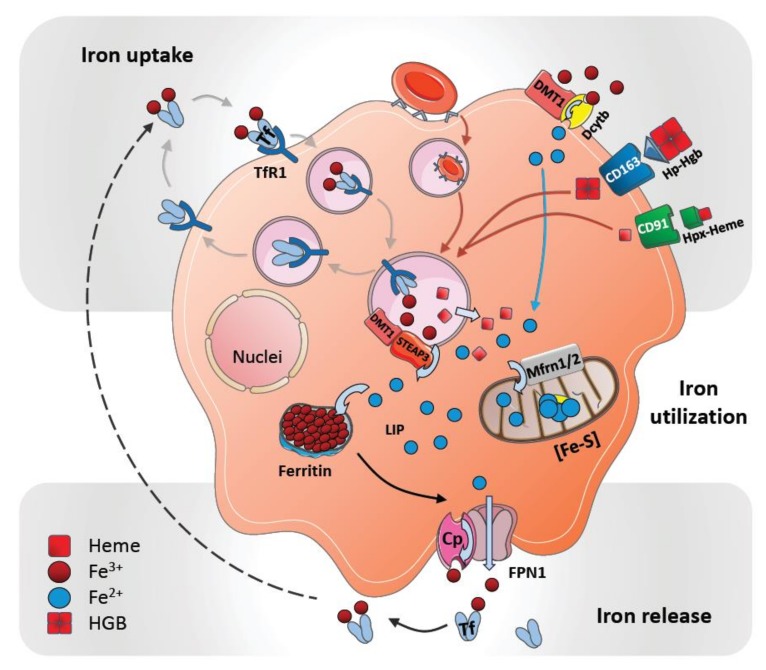
Iron metabolism by macrophages. General steps of iron uptake, acquisition, storage and release in the macrophage: In addition to iron extraction from red blood cells by erythrophagocytosis, macrophages express a variety of receptors to acquire iron from different sources. Transferrin-bound iron can be taken up by the transferrin receptor (TfR or CD71) and the complex is internalized by clathrin coated endocytosis followed by iron release at a low endosomal pH. Empty apo-transferrin and transferrin receptor complex are recycled again (grey arrows). Non-transferrin iron can be acquired directly via divalent metal transporter 1 (DMT-1) that is associated with duodenal cytochrome B (DcytB) (blue arrow). CD91 (LRP1) receptors scavenge hemopexin-bound heme (Hpx-Heme) iron, whereas haptoglobin- hemoglobin (Hp-Hgb) complex is taken up through CD163 receptor (red arrows). Iron transport across the endosomal membrane into the cytosol is accomplished through DMT1 after it is reduced by the endosomal reductase six-transmembrane epithelial antigen of the prostate 3 (STEAP3). The resulting intracellular free iron joins the labile iron pool (LIP), which is then either stored in ferritin or utilized in cellular processes for example in mitochondrial iron metabolism as ISC [Fe-S] after being transported through Mitoferrin 1/2 (Mfrn1/2). Iron can be exported through ferroportin 1 (FPN1) out of the cell, supported by the ferroxidase ceruloplasmin (Cp). Iron is then loaded to transferrin in order to be carried to the target cells.

Macrophages are important regulators of iron metabolism by controlling cellular iron import and export. While iron is taken up by macrophages in different forms, such as transferrin-bound iron, heme iron, Hgb-bound iron, and as free iron, iron egress from macrophages occurs only through ferroportin-1 (FPN1); encoded by the gene *SLC40A1* (Figure 1). 

Iron uptake mechanisms that are common to macrophages include the isolation of iron from senescent erythrocytes after phagocytosis; the import of transferrin-bound iron via transferrin receptor (TfR) or non-transferrin-bound iron (NTBI) via the divalent metal transporter 1 (DMT1), as well as zinc transporter ZIP14 (*SLC39A14*). Heme-bound iron can be taken up via different means. Heme-hemopexin (Hpx) complexes are internalized via its receptor CD91 (LDL receptor related protein 1, LRP1) and Hgb-bound iron in complex with its scavenger haptoglobin (Hp) is imported via its receptor CD163 (scavenger receptor cysteine-rich SRCR superfamily) (Figure 1). Additional pathways of iron transport into macrophages include natural resistance-associated macrophage protein 1 (NRAMP1/*SLC11A1*) and divalent cation transporter 1 (DCT1/NRAMP2) [9,10]. NRAMP1 is a divalent metal transporter that is expressed on the surface to mediate iron import, as well as within late endosomes and phagolysosomes to support intracellular iron recycling after erythrophagocytosis [11,12,13]. Other transporters that have been involved in cellular heme transportation include the heme-carrier protein 1 (HCP1) and the feline leukemia virus group C cellular receptor 1 (FLVCR1). HCP1 was first characterized as a cellular heme transporter, and later studies identified it as a proton-coupled folate transporter (PCFT/*SLC46A1*) [14,15]. Because HCP1 is abundantly expressed in macrophages and colocalizes with Hgb-Hp complexes, which are taken up via CD163, indeed suggest its dual role in cellular transport of heme and folate [16]. Besides implicated in heme uptake, other studies link FLVCR1 to cytoplasmic heme export that prevents intracellular heme overload [17,18]. Furthermore, heme responsive gene 1 (HRG1/*SLC48A1*) enables the transport of heme out of the phagolysosomes in erythrocyte-digesting macrophages. HRG1 is strongly expressed in macrophages and colocalizes with NRAMP1 to erythrophagolysosomes [19,20,21].

Ferritin is the main iron storage complex in cells (see below), but it can also be found in plasma. Two different ferritin receptors have been described that mediate uptake of ferritin-bound iron: Heavy chain H-ferritin (FtH) receptor T cell immunoglobulin and mucin domain 2 (TIM2) and light chain L-ferritin (FtL) receptor scavenger receptor, member 5 (Scara5) [22,23].

After uptake, iron is liberated from the various iron-containing proteins via different mechanisms (described below). Afterwards, ferrous iron (Fe^2+^) can be either translocated through the cytosol for further utilization in cellular processes, transported from the cell into the circulation or stored. In the cytosol iron becomes a part of a poorly described labile iron pool (LIP) [24,25]. It is assumed that the LIP directly triggers iron-utilizing cellular processes when cellular iron levels increase.

Excess iron is stored mainly in hepatocytes and macrophages as ferritin—a multimeric sphere-forming protein filled with up to 4500 iron atoms [26]. Ferritin consists of two distinct subunits: FtH and FtL [27]. Due to the ferroxidase activity of FtH, cytosolic ferrous iron (Fe^2+^) is oxidized into ferric iron (Fe^3+^) prior to storage in the ferritin cavity [28]. Furthermore, inside splenic macrophages an iron deposition complex known as hemosiderin can be found; these deposits form paracrystalline structures and are surrounded by intralysosomal formations. It has been interpreted as an aggregated form of partially digested ferritin, which may be only poorly available to supply iron when needed [29,30].

Iron within the cytosol of macrophages is utilized as a cofactor in many different cellular proteins that regulate energy production, hypoxic regulation, detoxification, and host defense and inflammation [31]. These proteins contain iron as part of two main cofactors: Iron-sulfur clusters (ISC) and heme groups. Both cofactors are synthesized in the mitochondria, where excess iron can also be stored as mitochondrial ferritin (FtMt). Iron is transported across the mitochondrial membrane in its ferrous form through mitoferrin 1 (Mfrn1) and Mfrn2 transporters [32]. Most complexes of the electron transport chain contain several ISC proteins that drive energy production. Important defense and inflammation-related proteins, such as myeloperoxidase, NADPH oxidase, indoleamine 2,3-dioxygenase, nitric oxide synthases or lipoxygenases that are produced by macrophages also contain iron. 

Under steady-state conditions, iron is released from the main iron stores by means of FPN1, the only known iron exporter known until now. FPN1 is abundantly expressed on all iron-metabolizing macrophages [2,33]. In individuals with an FPN1 mutation—as well as when FPN1 is inactivated genetically in mice—a macrophage-specific iron overload in the spleen and liver has been observed [34,35,36]. These observations suggest a macrophage-specific release of iron through FPN1. 

The function of FPN1 in iron-recycling macrophages is aided by a multicopper oxidase, ceruloplasmin (Cp, IREG1), which is synthesized and secreted by the liver and macrophages [37]. The transport of iron across all biological membranes is requires the oxidation and reduction of iron. Accordingly, different ferroxidases and ferroreductases are inevitable for iron transportation. Cp is commonly present on macrophage membranes and is thought to mediate iron oxidation to facilitate its export out of the cell by FPN1 [38]. Cp-deficient mice and humans with Cp mutations show hepatocyte- and macrophage-specific iron accumulation and develop anemia indicating a critical role of Cp in the process of iron release from macrophages [39,40,41]. Cp can be expressed both as a circulating plasma protein and as a membrane-bound GPI-linked protein [42]. Interestingly, FPN1 internalization and degradation are specifically performed in the absence of membrane-bound Cp; this may suggest that Cp is involved in hepcidin-mediated iron regulation [39]. 

After oxidation to ferric iron (Fe^3+^) and cellular egress, it is presumed that iron is loaded directly onto the glycoprotein transferrin, the main systemic iron transporter of ferric iron (Figure 1). Transferrin-bound iron is mostly utilized by developing erythrocytes and nurse macrophages in the BM to support erythropoiesis.

## 3. Systemic Iron Metabolism

Iron enters the body through epithelial cells of the duodenum and upper ilium in the small intestine. Iron absorption is adjusted to iron demands and can be increased to maintain iron requirements. The transfer of non-heme Fe^3+^ iron from the lumen of the duodenum into the epithelium is carried out by DMT1 (Figure 1).

Dietary ferric iron (Fe^3+^) is first reduced at the apical brush border of the epithelium to enter the enterocytes; this relies on the activity of iron-reducing ferric reductase duodenal cytochrome B (Dcytb or CYBRD1) [43]. DMT1 is colocalized with Dcytb at the apical side of enterocytes when absorption is elevated, and their expression is induced under iron-deficient conditions. This suggests that at the apical surface of the epithelium, DMT1 and Dcytb serve as main transport coordinators [44,45]. As an important source of dietary iron, heme containing ferrous iron (Fe^2+^) can also enter enterocytes through the heme transporter HCP1. 

Once taken up, iron in the cytosol of the enterocytes might be transferred from the apical to the basolateral site by means of vesicular trafficking [46]. An alternative mechanism is that iron transfer might be mediated by “chaperones”, such as the calreticulin-like protein mobilferrin [47]. 

The release of iron at the basolateral site of epithelium is facilitated by FPN1. The selective inactivation of FPN1 in the intestine results in a marked iron accumulation in duodenal enterocytes and systemic iron deficiency [48]. This confirms the essential function of FPN1 in the dietary iron release into the circulation [34,49]. Of particular relevance is the contribution of iron-oxidizing ferroxidases in FPN1-mediated egress of iron from the cellular environment. The membrane-bound ferroxidase hephaestin (HEPH), a closely related homolog to Cp, is presumed to be responsible for iron oxidation of FPN1-exported iron specifically in the enterocytes [50,51]. In addition, several studies have revealed a significant role of Cp for iron absorption in enterocytes under iron-stress conditions, such as bleeding and iron deficiency [52].

Interestingly, HEPH colocalizes with apo-transferrin in enterocytes [53]. This suggests a direct transfer of iron to transferrin after its release from the intestinal epithelium. Upon binding of two ferric iron (Fe^3+^) atoms, a holo-transferrin is formed, which enables a secure transport of ferric iron (Fe^3+^) through plasma. In healthy human individuals, approximately 30% of the total transferrin is saturated [33]. Holo-transferrin can bind to its receptor TfR (CD71) on macrophages, erythrocytes, and other cell types, which is then internalized through receptor-mediated endocytosis.

## 4. Systemic Iron Regulation by Hepcidin

**Figure 2:**
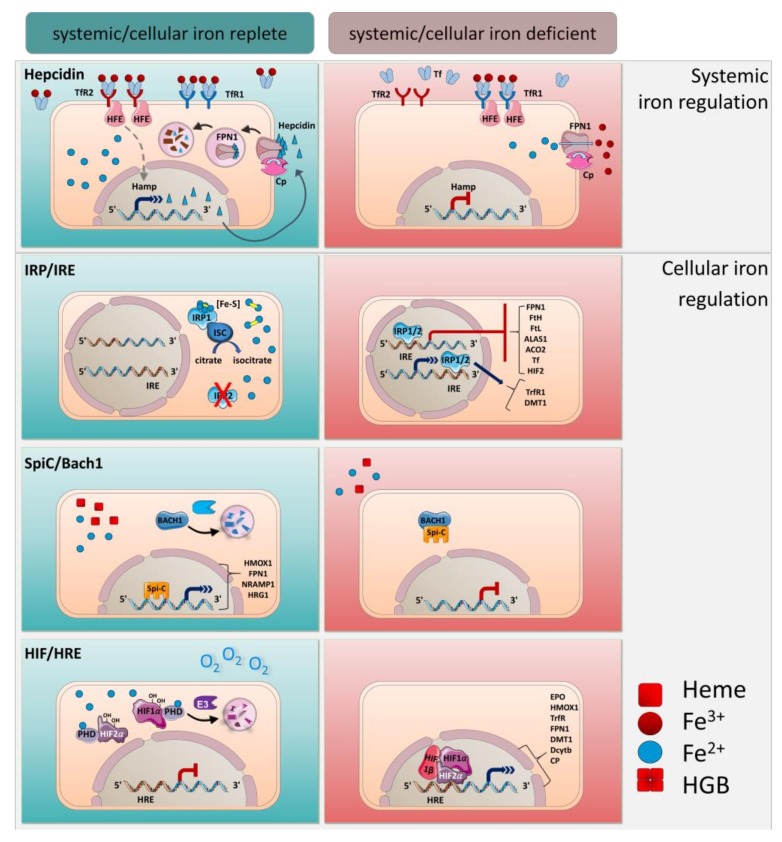
Main regulatory mechanisms of iron. Regulation of iron metabolism through hepcidin, the IRP/IRE, Spi-C/Bach1, and HIF/HRE. For details see text.

Iron homeostasis is regulated at the systemic and the cellular level by several well-coordinated mechanisms (Figure 2). Hepcidin is the main regulator of the systemic transportation of iron. It is a peptide hormone expressed mainly in the liver by hepatocytes and undergoes proteolytic processing to yield a bioactive molecule that is secreted into the bloodstream [31,54,55]. Hepcidin binds to FPN1 and mediates its phosphorylation, internalization and degradation. Thus, in FPN1-expressing cells, such as macrophages, hepatocytes, and duodenal epithelial cells, iron export is blocked [34,56,57]. 

Hepcidin expression is regulated by iron and iron-sensing mechanisms. Accordingly, the synthesis of hepcidin is increased by iron loading and decreased by anemia and hypoxia. In this manner, an increase of systemic transferrin iron leads to hepcidin expression that causes iron retention in the macrophages and blocks iron uptake in the enterocytes to avoid further systemic iron accumulation [34,56,58]. Inappropriate low hepcidin expression due to mutations in the hepcidin-encoding gene *Hamp* or mutations that impair hepcidin function are linked to hereditary hemochromatosis and total body iron overload [56,59,60,61,62]. On the contrary, pathological hepcidin excess caused by inflammatory disorders induces hypoferremia, anemia of inflammation, or anemia of chronic disease [31,63,64]. Therefore, hepcidin-mediated regulation is essential in maintaining iron homeostasis. 

Hepcidin expression is controlled transcriptionally by various signals, such as transferrin saturation, erythropoietic activity, hypoxia and inflammation, rendering it an important player in the process of iron homeostasis. Organisms have evolved multitude mechanisms to sense iron and to activate hepcidin expression (Figure 2). 

The molecular mechanism by which transferrin iron serves as a sensor for hepcidin expression is based on a coordinated interplay between TfR1 and TfR2 with the homeostatic iron regulator HFE, a major histocompatibility complex class I integral membrane protein [65,66]. Circulating transferrin-bound iron interacts with high affinity to TfR1, whereas TfR2 has a lower iron-binding affinity. Accordingly, under low transferrin saturation only TfR1 is engaged to transferrin iron. In this case only, HFE is able to associate with TfR1 and hepcidin expression is not induced, while TfR2 is directed to a lysosomal degradation pathway [1,66,67]. When transferrin iron levels increase in plasma, transferrin iron interacts also with TfR2, which leads to detachment of HFE from TfR1 and binding to TfR2 to induce hepcidin transcription [68,69,70]. Consequently, the competitive interaction between HFE and TfR1 or TfR2 determines iron regulation through hepcidin. In hemochromatosis patients with *HFE* gene mutations or in HFE-deficient mice, hepcidin synthesis is inappropriately low that induces systemic iron overload [65,71]. 

Furthermore, liver iron stimulates the bone morphogenetic protein (BMP) BMP6 signaling pathway to trigger hepcidin production [72,73]. Hepcidin induction by classical BMP signaling is dependent on the BMP co-receptor hemojuvelin (HJV or HFE2), a GPI-linked membrane protein. HJV binds to BMP6 and activates the Smad family of transcription factors to stimulate *Hamp* transcription [58].

Inflammatory mediators are major hepcidin-inducing factors. Lipopolysaccharide, interleukin-6 (IL-6), IL-1, or IL-22 induce hepcidin partially via signal transducer and activator of transcription 3 (STAT3) [64,74,75,76]. Hepcidin expression in response to infections rapidly lowers plasma iron to prevent iron sequestration of microbes that need iron for growth [77]. Additionally, macrophages are able to produce hepcidin locally in response to bacterial signals at the side of the infection to limit iron bioavailability for pathogens [78,79]. Thus, iron regulation by macrophages is an important defense mechanism during an infection [80].

On the contrary, hepcidin expression is blocked by hypoxia to stimulate iron mobilization and erythropoiesis [81,82]. Other hepcidin inhibitory factors were identified, including members of the growth factor beta (TGF-β) superfamily comprising growth differentiation factor 15 (GDF15), transforming growth factor beta (TGF-β), twisted gastrulation BMP signaling modulator 1 (TWSG1) and erythroid factor erythropoietin (Epo) [83,84].

## 5. Cellular Regulation of Iron by IRE/IRPs

Compared to the transcriptional regulation of hepcidin, many proteins involved in controlling intracellular iron metabolism are governed by posttranscriptional mechanisms. The main cellular iron uptake, transport, storage, utilization, and release processes are governed by the interplay of distinct mRNA-binding iron-regulatory proteins (IRPs), which have been extensively studied in macrophages and hepatocytes [1,85]. Two different IRPs are known: IRP1 and IRP2. IRPs bind to their target transcripts that contain cis-regulatory iron-regulatory elements (IREs), conserved RNA stem loop structures [86,87,88]. Besides iron status, the localization of IREs on transcripts determines whether a given IRP-IRE interaction stimulates or suppresses protein translation (Figure 2). 

In cellular iron deficiency, IRPs bind to IREs at the 5’ untranslated region (UTR) of FPN1, FtH, FtL, delta-aminolevulinate synthase 1 (ALAS1), aconitase 2 (ACO2), hypoxia-inducible factor 2 (HIF2) mRNAs to mediate the degradation of the transcripts of the IREs to decrease iron storage and export [89]. In addition, the binding of IRPs to IREs at 3’ UTR of TfR1 and DMT1 mRNA stabilizes these transcripts and supports their translation to increase iron import [37,85]. 

Conversely, under cellular iron-replete conditions, IRP2 gets ubiquitinated and degraded by the proteasome to inhibit binding to IREs [90,91,92,93]. IRP1 similarly loses its IRE-binding activity but acquires an ISC [4Fe-4S] to obtain cytosolic aconitase activity that converts citrate to isocitrate in the citric acid cycle [94]. Because the bi-functional IRP1 shifts between iron-regulation and metabolism-regulation, this mechanism suggests an interesting link between iron and cellular energy metabolism [95,96,97,98]. IRP1^−/−^ and IRP2^−/−^ mice show massive impaired iron homeostasis suggesting that the IRP/IRE system is critical in cellular iron control [95,98,99].

## 6. Transcriptional Regulation by Spi-C and HIF

Iron homeostasis on the cellular level is also regulated transcriptionally by the induction of the transcription factors Spi-C and HIFs. Endogenous labile heme derived from e.g., hemolysis binds to and liberates Spi-C from its repressor BTB domain and CNC homolog 1 (BACH1), which is subsequently degraded by the ubiquitin−protein ligase HOIL-1 [100,101,102,103]. Unbound Spi-C can enter the nucleus and directly stimulates important heme- and iron-regulating proteins, such as heme oxygenase 1 (HMOX1), FPN1, NRAMP1, and HRG1. Spi-C-mediated control of iron genes in macrophages has been reported to be particularly important when rapid iron metabolism is needed; for example, through increased erythrophagocytosis or hemolysis [102,104]. Iron-driven Spi-C activation is a critical factor for the development of iron-specific macrophages. Moreover, the number of BM macrophages and RPMs is drastically reduced in *Spic*^−/−^ mice suggesting that Spi-C-mediated regulation is indispensable for iron metabolism in these cells and for protection against iron toxicity [100,105].

Additionally, iron metabolism is regulated in response to oxygen by means of HIF1α and HIF2α. If sufficient levels of ferrous iron (Fe^2+^) and oxygen are present, HIF1α and HIF2α are hydroxylated by oxygen prolyl hydroxylases (PHDs) and are then degraded by Ligase E3 [106,107]. In contrast, low iron levels in cells, as well as hypoxia, induce an accumulation of HIF1α and HIF2α in the nucleus and subsequently triggers the recruitment of HIF1β. Accordingly, the HIFα/HIFβ complex binds to hypoxia-responsive elements (HRE) and promotes transcription of genes that are involved in iron metabolism such transferrin, TfR, DMT1, FPN1, Dcytb, Cp, and HMOX1 [108,109]. In line, the HIF1/2-HRE interaction in response to hypoxia and to low iron induces iron release from macrophages [110,111]. This process is not only important in macrophages, but also induces RBC generation and maturation through Epo [112]. HIF2α contains IREs in its 5’ UTR of mRNA and is thus under control of IRP1. These findings show that iron homeostasis depends on complex, global and cellular regulations at various levels of gene expression.

## 7. Steady-State Erythrophagocytosis by Macrophages in the Spleen

The majority of iron (20–21 mg per day) is recycled from heme of erythrocytes in the spleen, whereas only 1–2 mg of iron is absorbed by the diet. Macrophages extract iron from senescent erythrocytes by disassembling Hgb. Approximately 1 billion iron atoms are extracted from 250–280 million Hgb molecules per erythrocyte [113]. Thus, almost all iron atoms pass through macrophages for further cellular and systemic utilization.

**Figure 3:**
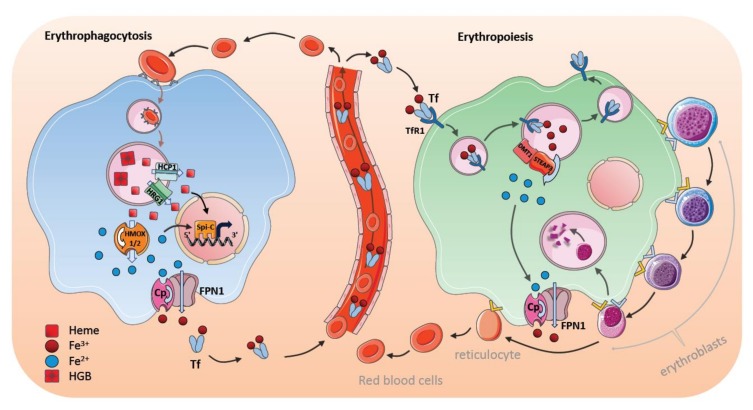
Macrophages in erythrophagocytosis and erythropoiesis. The majority of iron in a macrophage is obtained during erythrophagocytosis. Splenic red pulp macrophages (RPMs) and liver Kupffer cells (KCs) is the main source of iron in the body. Senescent red blood cells (RBCs) are recognized by macrophages and digested in the lysosomes. Heme is extracted in the phagolysosomes and transferred to the cytosol by heme transporters heme-carrier protein 1 (HCP1) and heme responsive gene 1 protein (HRG1). Heme degradation by heme oxygenase 1 (HMOX1) in the cytosol provides ferrous iron that enters the intracellular labile iron pool. Iron is either stored in ferritin or exported by ferroportin 1 (FPN1) and ceruloplasmin (Cp). The transcription factor (Spi-C) protects iron processing RPMs in the spleen and BM nurse macrophages. Central nurse macrophages in the bone marrow (BM) promote erythropoiesis in the erythroblastic island niche. These macrophages ubiquitously express transferrin receptors and thus, probably mainly utilize transferrin (Tf)-bound iron. Nurse macrophages are important for the development of erythroblasts. This is mediated by adhesion molecules that control proliferation and differentiation of erythroblasts, enable erythroblast nuclei ingestion, and control the subsequent release of reticulocytes into the blood stream. Central macrophages are supposed to be involved in supplying maturing erythrocytes with iron to trigger heme synthesis.

The first signal that stimulates iron digestion in macrophages is the recognition of senescent RBCs and the uptake of these cells by means of receptor-mediated erythrophagocytosis (Figure 3). The macrophage surface is equipped with various scavenging receptor proteins to recognize the “eat me” signals from aged RBCs [114,115]; these include, among others, signal-regulatory protein alpha (SIRPα), glucose-6-phosphate dehydrogenase, phosphatidylserine (PS) receptors (such as TIM4 and TIM1), scavenger receptor type A member I (SR-AI), and CD36. The most popular presenters of the “eat me” signals are CD47 and PS, which are exposed to aging RBCs [10,116,117]. After internalization, RBCs-containing phagosomes merge with lysosomal vesicles to form phagolysosomes, where the RBCs are digested. Hgb breaks down into heme and heme is then transferred from the erythrophagolysosomes into the cytosol [21,118]. The transport of heme out of the erythrophagolysosome is facilitated by a heme transporter, known as HRG1, which is regulated posttranscriptionally by heme and iron [19]. HRG1 expression is high in the macrophages of the spleen, liver, as well as BM, and it localizes in erythrophagolysosomes one hour after erythrophagocytosis. In addition, the putative heme transporter HCP1 has been suggested to transport heme into the cytosol [16]. In the cytosol of macrophages, iron is immediately processed from the protoporphyrin ring of heme by means of HMOX1 [119]. Degradation of heme by HMOX1 generates equivalent amounts of CO and biliverdin in addition to iron [120]. HMOX1 is not present on the erythrophagocyte membrane, but has been located in the endoplasmic reticulum with its active site directed to cytosol; this process supports the idea that iron is extracted in the cytosol [19,121]. Moreover, HMOX1 expression can be detected immediately after erythrophagocytosis or after hemolysis corroborating its crucial role in iron and heme detoxication [122,123]. The role of HMOX1 is underscored by the finding that HMOX1 deficiency causes a depletion of RPM and BM macrophages; suggesting that the survival and function of iron-metabolizing macrophages is dependent on HMOX1. In addition, there is a second heme oxygenase HMOX2, which is involved in removing intracellular heme. In contrast to HMOX1, which is inducible, HMOX2 is continuously expressed in most tissues and is involved in extracting iron in different cell types [122,123]. 

Recently, NRAMP1 (SLC11A1) has been identified as an important iron transporter in the macrophages, which is localized to erythrophagolysosomes after erythrophagocytosis [21,124]. NRAMP1-knockout mice retain iron in the macrophages, but release storage iron from hepatocytes to compensate for the impaired iron efflux from macrophages [124]. 

Depending on erythropoietic activity and other iron-consuming processes, iron can be either stored or released through FPN1, and its expression increases in macrophages within an hour after erythrophagocytosis [49,125]. Moreover, erythrophagocytosis-activated macrophages have concomitant high levels of heavy (H-) and light (L-)ferritin content, even several hours after erythrophagocytosis has occurred; this indicates that a substantial proportion of iron can be stored intracellularly within ferritin after erythrophagocytosis in the liver and the spleen [29,126]. An increase of cellular iron upon systemic hemolysis, when iron storage in KCs of the liver is saturated, iron has been found to be transferred into hepatocytes for long-term storage [127].

## 8. Stress-Induced Erythrophagocytosis and Iron Metabolism

Under steady-state conditions, heme iron is preferentially digested by RPMs in the spleen, whereas, under stress conditions, macrophages in the liver become essential for erythrophagocytosis. For example, during systemic hemolysis, Ly6C^+^ monocytes migrate to the liver and, thereupon, differentiate into iron-recycling macrophages, but not to KCs [127]. Interestingly, colony stimulating factor 1 (CSF1) and nuclear factor erythroid 2-related factor 2 (Nrf2) transcription factor promote the development of these iron-recycling FPN1^+^Tim4^neg^ macrophages from blood-borne monocytes in the liver [127]. Moreover, iron recycling by RPMs in the spleen is not ameliorated to compensate for the increased iron need. Hence, FPN1^+^Tim4^neg^ iron-recycling liver handle and recycle heme and iron from senescent and damaged RBCs after massive hemolysis [10].

Macrophages have developed further elaborate mechanisms to remove systemic heme during hemolysis. The body responds immediately against toxic free Hgb and heme by producing the Hgb-scavenging protein haptoglobin and the heme-binding serum protein hemopexin. Splenic RPMs and liver KCs express the receptors CD163 and CD91: these are involved in the clearance of excessive labile heme and, importantly, offer an acute protection mechanism [128,129]. Macrophages possess ferroxidases (such as Cp and HEPH) and proteins promoting ferrireductase activity (such as FtH and Dcytb); these enable the quick oxidation and reduction of iron, and ensure efficient transportation and utilization of iron within a cell [130]. Additionally, as described above, heme-mediated Spi-C induction in monocytes promotes the differentiation of erythrophagocytosing RPMs in the spleen and induces the expression of important heme- and iron-regulating proteins [100]. This facilitates a quick heme and iron processing and inhibits iron-induced toxicity in the cells [21,29,100,101] (Figure 3).

Recently, it was demonstrated that labile heme released after massive hemolysis, following bacterial infection, suppresses the phagocytosing function of macrophages [131]. It is conceivable that the liberation of heme when erythrophagocytosis commences may function as a negative feedback loop and impairs further erythrophagocytosis by macrophages as a protective mechanism to sustain oxygen supply.

## 9. Bone Marrow Macrophages and Erythroblastic Islands

Erythropoiesis is a dynamic process that is regulated through environmental signals, nutrient availability, and cellular interplay. Most of the transferrin-bound iron is utilized for heme synthesis during definitive erythropoiesis in the BM. In the BM, CD169 (Sialoadhesin or Siglecß1) is expressed on certain macrophages—referred to as nurse macrophages. These form an erythroblastic island, in which the central nurse macrophage is surrounded by erythroid progenitors at different developmental stages and provides a niche for erythropoietic regulation [132,133,134] (Figure 3). Thus, the nursing macrophage supports erythropoiesis at different stages through varying the stimulating and repressing signals to control proliferation versus differentiation of erythroid progenitors, and to sustain survival signals [134,135]. Depending on the developmental stage, different soluble factors, such as BMP4, insulin-like growth factor 1 (ILGF1), IL-3, and granulocyte-macrophage colony-stimulating factor (GM-CSF) are secreted from the central nurse macrophages and induce proliferative and differential signals in erythroid progenitors [136,137]. In addition, negative regulators of erythropoiesis can be released by BM macrophages, including GATA binding protein 1 (GATA1), TGF-β, interferon gamma (IFNγ), and tumor necrosis factor alpha (TNFα) [136,138].

Central nurse macrophages are thought to be involved in phagocytosing the extruded nuclei from erythroblasts during their development. This process is based on recognition through PS exposure, TIM4 and MER proto-oncogene tyrosine kinase (MerTK) [2,139]. Because developing erythroblasts adhere to the central macrophage, it has also been hypothesized that BM macrophages regulate and determine the release of mature reticulocytes into the blood circulation. Prominent adhesion molecules that are involved in cell–cell interaction, signal transduction, and reticulocyte release controlled by macrophages include erythroblast macrophage protein, vascular cell adhesion protein 1 (VCAM1) and a4b1- or a4b5-integrins [136,137,140]. Ablation of macrophages, or one of the adhesion molecules, delays proerythroblast proliferation and differentiation at different developmental stages and to destroy the erythroblastic island [136,137,141]. Moreover, Spi-C-mediated signals are also essential for erythropoiesis, since Spi-C ablation causes a reduction in erythroblastic island numbers and a delay in RBC formation [100]. Interestingly, CD169+ macrophage ablation, which markedly reduces the number of erythroblasts in the bone marrow, only leads to a mild reduction of erythroid cells without overt anemia suggesting that erythropoietic compensatory mechanisms exist during homeostasis [141]. However, the induction of stress-induced anemia (for example, through phenylhydrazin treatment) promoted more severe delays in erythropoietic recovery in CD169^+^-ablated animals. This indicates that BM macrophages promote fast recovery responses to stress-induced erythropoiesis [132]. On the contrary, pathological erythropoiesis in mouse models of polycythemia vera (PV) can be reversed through the ablation of central macrophages [142].

## 10. Regulation of Iron Transfer by Central Nurse Macrophages

Transferrin-bound iron is the main iron source for erythropoiesis. Iron-bound transferrin and its receptor are internalized by receptor-mediated endocytosis [143]. TfR expression on erythroblasts increases during erythrocyte development, but is downregulated after the release of reticulocytes [144]. Loss of transferrin impedes erythropoiesis and leads to an accumulation of iron in the storage organs; this indicates the importance of transferrin-bound iron as a crucial source for erythropoiesis [145]. BM nurse macrophages contribute to iron-loading of the erythroblasts, which might be especially pronounced during stress conditions. In the macrophage, iron atoms are released from transferrin in the acidic condition within endosomes (pH 4.5), and transferrin, along with TfRs, is recycled back onto the macrophage surface. Ferric irons (Fe^3+^) in the endosomes are reduced to ferrous irons (Fe^2+^) by six-transmembrane epithelial antigen of the prostate 3 (STEAP3)—an endosomal ferrireductase located in endosomes [146]. Ferrous iron (Fe^2+^) is then transported by means of DMT1 through the endosomal membrane and is released into the cytosol. It is not entirely clear how iron is subsequently translocated through the cellular cytosol and utilized for erythropoiesis. The high expression of FPN1 on the nurse macrophages and the observation that FPN1 deletion leads to an accumulation of iron in BM macrophages and a blockage of erythroblast development suggest that iron is released from macrophages prior to being loaded into the erythroblasts [147,148]. It is also possible that NRAMP1, which participates in iron transport in BM macrophages, is responsible for FPN1-associated iron release during erythropoiesis [124,149]. Interestingly, in the absence of transferrin-bound iron, macrophages utilize other forms of iron, such as secreted ferritin iron and heme iron to support erythropoiesis [150,151]. However, the importance of these alternative pathways in macrophage-dependent iron transfer remains to be determined.

## 11. Concluding Remarks

Efficient iron metabolism is a matter that decides between life and death and depends on complex parallel, compensatory, and complementary networks to meet the iron demand for erythropoiesis, as well as other cellular functions, but to prevent iron overload, which cause cellular and organismal toxicity. Tissue macrophages take center stage in iron homeostasis and their regulatory mechanisms are increasingly recognized. Except duodenal iron uptake, macrophages are implicated in every step of iron recycling and metabolism. Future studies may identify new functions of macrophages that regulate tissue-specific iron metabolism and contribute to metabolic disorders. One emerging theme from knock-out mice suggests that macrophage-mediated iron metabolism is particularly important during stress conditions. Whereas these mouse models show that under homeostatic conditions, compensatory mechanisms exist that maintain systemic iron metabolism, macrophages may still be the main iron regulators in unaltered wild-type mice. Evidence implicates the importance of the central macrophages in erythropoietic regulation, however, it is not known how macrophages sense erythropoietic need and whether macrophages coordinate erythropoiesis dependent on iron availability and oxygen demand. The sensing of these factors is essential for the proper regulation of RBC maturation. Thus, HIF-regulatory mechanisms may be an attractive candidate, because they can sense both iron and oxygen. The understanding of cellular and systemic regulation of iron homeostasis will help to develop novel therapeutic possibilities in anemic diseases and other conditions, such as malaria, acute blood loss, hematopoietic stem cell transplantation, or chronic kidney disease.

## Abbreviations

ACO2	aconitase 2
ALAS1	delta-aminolevulinate synthase 1
BACH1	BTB domain and CNC homolog 1
BM	bone marrow
BMP	Bone morphogenetic protein
Cp	ceruloplasmin
DCT1	divalent cation transporter 1
DcytB	duodenal cytochrome B
DMT1	divalent metal transporter 1
E3	E3 ubiquitin ligase
Epo	erythroid factor erythropoietin
FLVCR1	Feline leukemia virus group C cellular receptor 1
FPN1	ferroportin 1
FtMt	Mitochondrial ferritin
FtH	heavy chain H-ferritin
FtL	light chain L-ferritin
GDF15	growth differentiation factor 15
GM-CSF	granulocyte-macrophage colony-stimulating factor
*Hamp*	hepcidin antimicrobial peptide
HEPH	hephaestin
HCP1	heme-carrier protein 1
*HFE*	homeostatic iron regulator
Hgb	hemoglobin
HIF1α/HIF2α	hypoxia-inducible factor 1α/2α
HJV	hemojuvelin
HMOX1	heme oxygenase 1
Hp	haptoglobin
Hpx	hemopexin
HRE	hypoxia-responsive element
HRG1	heme responsive gene 1 protein
ILGF1	insulin-like growth factor 1
IFNγ	interferon gamma
IRE	iron-regulatory element
IRP1/IRP2	iron-regulatory protein1/2
ISC	iron-sulfur cluster
KC	Kupffer cell
LIP	Labile iron pool
Mfrn1/2	Mitoferrin 1/2
MerTK	MER proto-oncogene tyrosine kinase
NTBI	non-transferrin-bound iron
NRAMP1	natural resistance-associated macrophage protein 1
PHDs	oxygen prolyl hydroxylases
PS	phosphatidylserine
PV	polycythemia vera
RBC	red blood cell
RPM	red pulp macrophages
SIRPα	signal-regulatory protein alpha
Spi-C	Spi-1/PU.1 related transcription factor
SR-AI	scavenger receptor type A member I
STAT3	activator of transcription 3
STEAP3	six-transmembrane epithelial antigen of the prostate 3
Tf	transferrin
TfR1/2	transferrin receptor 1/2
TNFα	tumor necrosis factor alpha
TGF-β	transforming growth factor beta
TWSG1	twisted gastrulation BMP signaling modulator 1
VCAM1	vascular cell adhesion protein 1
UTR	untranslated region
